# Use of Glucagon-Like Peptide-1 (GLP-1) Agonists in Modulating Preexisting Dermatologic Disease: A Systematic Review

**DOI:** 10.7759/cureus.93282

**Published:** 2025-09-26

**Authors:** Bryan Tassavor, Sultan Al Salem

**Affiliations:** 1 Osteopathic Medicine, A.T. Still University, Mesa, USA; 2 Dermatology, King Saud University, Riyadh, SAU

**Keywords:** anti-inflammatory therapy, dermatologic disease, glp-1 receptor agonists, metabolic syndrome in dermatology, weight loss in dermatology

## Abstract

Glucagon-like peptide-1 receptor agonists (GLP-1RAs) have transformed the management of type 2 diabetes and obesity, and growing evidence suggests potential benefits in dermatologic disease. We systematically reviewed reports in the literature that linked their use to improvement of preexisting dermatologic conditions, including psoriasis, hidradenitis suppurativa, hyperandrogenism, insulin resistance, and acanthosis nigricans (HAIR-AN) syndrome, Hailey-Hailey disease, acne keloidalis nuchae, folliculitis decalvans, androgenic alopecia, and localized linear scleroderma. Through controlled trials, cohort studies, and case reports, varying degrees of clinical improvement were observed across these conditions. We also review proposed mechanisms underlying these findings, with attention to immunologic, metabolic, and barrier-modulating pathways. GLP-1RAs thus represent a promising therapeutic avenue in dermatology, warranting further investigation in larger, prospective studies.

## Introduction and background

Glucagon-like peptide-1 receptor agonists (GLP-1RAs) are anti-obesity and anti-diabetes medications that mimic the action of the endogenous incretin hormone GLP-1, which is released from the gut in response to food intake. These agents enhance glucose-dependent insulin secretion, suppress glucagon release, slow gastric emptying, and increase satiety, thereby improving glycemic control and promoting weight loss [1].

Beyond their metabolic benefits, GLP-1RAs have drawn attention in dermatology because obesity, insulin resistance, and systemic inflammation are recognized contributors to chronic skin disease. In addition, GLP-1RAs appear to influence inflammatory and immune pathways, suggesting their effects may extend to dermatologic conditions. This overlap between metabolic and inflammatory mechanisms provides a strong rationale for investigating their therapeutic role in skin health.

Against this background, we present a systematic review of reports on GLP-1RA use in dermatology and summarize the range of preexisting skin conditions reported to be modulated following their initiation.

## Review

Methods

This systematic review followed the 2020 Preferred Reporting Items for Systematic Reviews and Meta-Analyses (PRISMA) guidelines. PubMed, CINAHL Plus with Full Text, MEDLINE Complete, and the Cochrane Library were searched from 2015 through 2025, with the final search on March 4, 2025, using the Boolean strategy: ([GLP-1] OR [Semaglutide]) OR [Dulaglutide] OR [Exenatide] OR [Liraglutide] OR [Lixisenatide] OR [Tirzepatide] OR [Albiglutide]) AND ([skin] OR [dermatology] OR [dermatologic] OR [cutaneous]). 

Eligible studies included full-text case reports, clinical trials, and cohort studies. Exclusions were review articles, basic science studies, duplicates, non-human studies, reports not addressing dermatologic disease, those in which GLP-1RAs were not an active intervention, studies focused only on side effects in otherwise healthy patients, and articles published more than 10 years ago. Studies were grouped by design, dermatologic condition, and GLP-1 receptor agonist.

Two reviewers independently screened records and full texts; discrepancies were resolved by consensus. Rayyan (Qatar Computing Research Institute, Doha, Qatar) was used for duplicate removal and to assist initial screening. Data extraction was also performed independently by two reviewers using a standardized form.

Extracted items included primary outcomes such as Psoriasis Area and Severity Index (PASI), Dermatology Life Quality Index (DLQI), and investigator-reported clinical improvement, and secondary variables including patient demographics, dermatologic condition, GLP-1RA type, dose, duration, weight change, biomarkers, and adverse effects. Missing or unreported data were coded as not reported. Effect measures were summarized as presented in the original studies: mean or median changes for continuous outcomes, proportions for categorical outcomes, and narrative descriptions for case reports.

Risk of bias was assessed by study type: randomized trials with Cochrane RoB 2, cohort studies with the Newcastle-Ottawa Scale, and case reports with the Joanna Briggs Institute checklist. Two reviewers conducted assessments independently and resolved disagreements by consensus.

**Figure 1:**
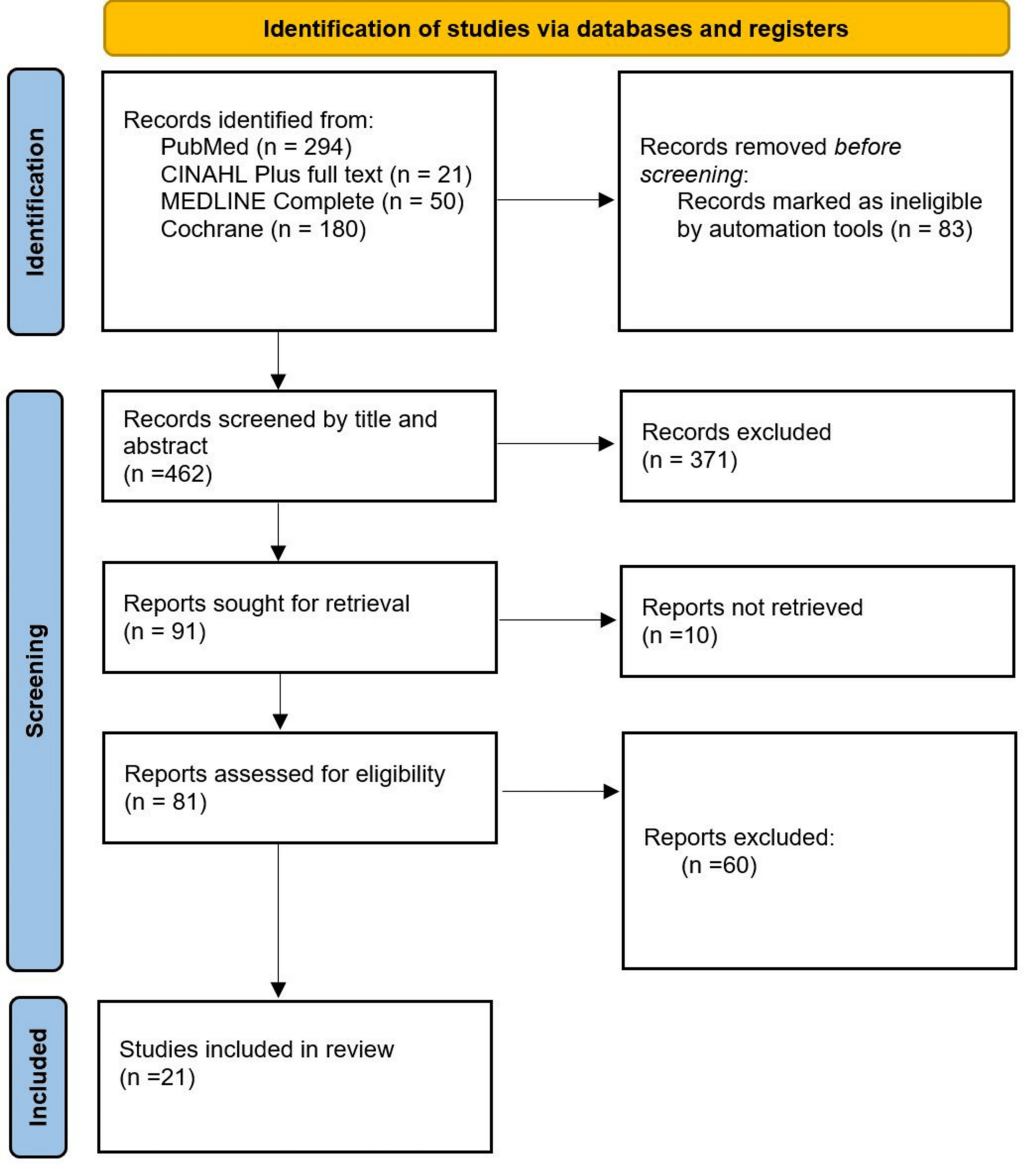
PRISMA flow diagram of study selection. The image is created by the author.

Due to heterogeneity in study designs and outcomes, findings were narratively synthesized and tabulated by study design, dermatologic condition, and GLP-1RA used. Meta-analysis and sensitivity analyses were not feasible. Certainty of evidence was not graded, given the predominance of case reports and variable outcome reporting. Study selection is shown in the PRISMA flow diagram (Figure 1).

Literature review

The literature review extracted eight psoriasis studies, six HS studies, two hyperandrogenism, insulin resistance, and acanthosis nigricans (HAIR-AN) studies, one benign familial pemphigus (Hailey-Hailey disease [HHD]) study, one acne keloidalis nuchae (AKN) study, one androgenic (AGA) study, one folliculitis decalvans (FD) study, and one congenital linear scleroderma (CLS) study. The results of the search are summarized in Table 1.

**Table 1:** A chronological summary of the findings in the relevant literature associating GLP-1 agonist use with modulating prior dermatologic disease. Study findings were organized by article type, preexisting dermatologic condition, GLP-1 agonist used, treatment regimen, and treatment outcome. PASI: Psoriasis Area and Severity Index, BMI: Body Mass Index, HS: Hidradenitis Suppurativa, T2DM: Type 2 Diabetes Mellitus, HAIR-AN: Hyperandrogenism, Insulin Resistance, and Acanthosis Nigricans, DLQI: Dermatology Life Quality Index, PCOS: Polycystic Ovary Syndrome, GLP-1RA: Glucagon-like Peptide-1 Receptor Agonist, AKN: Acne Keloidalis Nuchae, AGA: Androgenic Alopecia, HbA1c: Hemoglobin A1c, CRP: C-Reactive Protein, HOMA-IR: Homeostatic Model Assessment of Insulin Resistance, VAS: Visual Analog Scale, HS-PGA: Hidradenitis Suppurativa–Physician's Global Assessment, HiSCR: Hidradenitis Suppurativa Clinical Response, DAPSA: Disease Activity Index for Psoriatic Arthritis, ESR: Erythrocyte Sedimentation Rate, CI: Confidence Interval, RR: Risk Ratio, CLS: Congenital Linear Scleroderma, MTX: Methotrexate, MMF: Mycophenolate Mofetil.

Study	Article Type	Subject Details	Treated Condition	GLP-1 Agonist Used	Regimen	Outcome
Faurschou et al. (2015) [2]	Randomized-controlled trial	20 obese patients with a Psoriasis Area and Severity Index (PASI) of at least 8 without any active treatment within the last 3 months, a 3-month period with a body mass index (BMI) >25 kg/m 2 and no diabetes diagnoses.	Psoriasis	Liraglutide	Control group: 0.6 mg subcutaneous Liraglutide placebo for 1 week, 1.2 mg the following week and 1.8 mg for the remaining treatment period. Total treatment time: 8 weeks Experimental group: 0.6 mg subcutaneous Liraglutide for 1 week, 1.2 mg the following week and 1.8 mg for the remaining treatment period. Total treatment time: 8 weeks	No significant difference in PASI score (p = 0.228) or Dermatology Life Quality Index (DLQI) scores (p=0.564). Patients had significantly greater weight loss with liraglutide than with placebo (−4.7 ± 2.5 kg vs −1.6 ± 2.7 kg, p = 0.014). One patient developed a rash and was excluded from the study.
Jennings et al. (2017) [3]	Case Report	31-year-old female with obesity, Hurley stage II HS lesions on submammary, abdomen, groin and upper thigh with multiple prior treatment failures, including biologics.	Hidradenitis suppurativa	Liraglutide	0.6 mg subcutaneously daily, titrated to 1.8 mg daily over 8 weeks.	Resolution of submammary, abdomen and groin lesions with minimal remaining upper thigh lesions. Physician’s Global Assessment (HS-PGA) score reduced from 4 to 1 and DLQI score reduced from 24 to 14. Patients experienced a total weight loss of 6.5 kg, with no reported change in Homeostatic Model Assessment of Insulin Resistance (HOMA-IR). The patient reported a decreased need for narcotics.
Xu et al. (2019) [4]	Prospective Cohort Study	7 patients with T2DM with a mean age of 60 years with a psoriasis diagnosis of mean duration of 6 years. The patients were not on any medications for psoriasis	Psoriasis	Liraglutide	0.6 mg subcutaneously once daily for 1 week followed by 1.2 mg daily the next week and then increased to 1.8 mg daily. Total treatment time: 12 weeks.	Treatment reduced PASI from 15.7 to 2.0 (p < 0.05) and DLQI from 22 to 4 (p < 0.01). Clinical examination showed marked lesion improvement across multiple sites, with histology confirming resolution of psoriatic features. BMI decreased from 23 to 21 kg/m² (p < 0.01), and HOMA-IR declined from 2.8 to 1.6 (p = 0.03).
Livadas et al. (2020) [5]	Prospective Cohort Study	5 women with HAIR-AN. The subjects were of a mean age of 29 ± 2.4 years and mean BMI of 31 ± 3.5 kg/m2. Patients had not used metformin within 3 months of the trial.	HAIR-AN	Liraglutide	1.8 mg per day subcutaneously for a mean duration of 14 ± 4 months	Improvement of menstrual cycles, hirsutism and acanthosis nigricans lesions noted. Mean BMI dropped from 31 to 30, insulin dropped from 39.25 to 22.44, HOMA-IR dropped from 8.388 to 5.118 and testosterone dropped from 1.02 to 0.63.
Costanzo et al. (2021) [6]	Case Report	73-year-old male with obesity, T2DM uncontrolled by metformin and psoriasis.	Psoriasis	Semaglutide	0.25 mg subcutaneously for 4 weeks which was increased to 0.50 mg per week. By 16 weeks the regimen was 1 mg once weekly. Total treatment time: 10 months.	PASI score reduced from 33.2 to 2.6 and DLQI score reduced from 26 to 0. Psoriatic lesions on upper and lower limbs were subjectively reported to be greatly improved. BMI reduced from 40.3 to 38.3.
Lin et al. (2022) [7]	Randomized-controlled trial	24 patients with T2DM and psoriasis between 18-75 years old.	Psoriasis	Liraglutide	Control group: Acitretin (30-50 mg/day), Calcipotriol ointment and metformin or another oral hypoglycemic agent. Total treatment time: 12 weeks Experimental group: Acitretin (30-50 mg/day), Calcipotriol ointment, metformin or another oral hypoglycemic agent and Subcutaneous liraglutide injections for up to 1.8 mg weekly. Total treatment time: 12 weeks Note on metformin use in trial: 3 patients took metformin 2000 mg/day, 4 of patients took metformin 1000 mg/day + glimepiride 2 mg/day and 4 took metformin 1000 mg/day + sitagliptin 100 mg/day.	Control group: PASI decreased from a mean of 13.57 to 7.42, DLQI decreased from a mean of 18.23 to 9.6. The observed amount of cell layers on histopathologic analysis decreased by 3.93. On immunohistochemical staining of skin tissue samples, levels of IL-17, IL-23, and TNF-α were noted to have decreased significantly. BMI increased by 0.69 kg/m² and HOMA-IR rose by 0.37. Experimental group: PASI decreased from a mean of 14.02 to 2.40, DLQI decreased by a mean of 22.00 to 3.82. The observed amount of cell layers on histopathologic analysis decreased by 8.48. On immunohistochemical staining of skin tissue samples, levels of IL-17, IL-23, were noted to have decreased significantly in comparison to the control group with no significant difference in the decrease in TNF-α level compared to the control group. BMI decreased by 1.77 kg/m² and HOMA-IR decreased by 0.94 Compared results: There was a significant decrease in PASI (p=0.049), DLQI (p<0.001) and observed cell layers (p = 0.025). There was a significant improvement in BMI (p < 0.001) and a nonsignificant reduction in HOMA-IR (p = 0.074).
Drumm et al. (2023) [8]	Case report	21-year-old female with obesity, Hurley stage II HS and failed treatment courses of antimicrobials, metformin, spironolactone and adalimumab	Hidradenitis suppurativa	Semaglutide	1 mg subcutaneously once weekly for 6 months	DLQI score and other subjective clinical scores improved. The patient was noted to have lost 24 kg and reported no further flares at the end of the treatment period with no further need for analgesics.
Li et al. (2023) [9]	Case Report	11-year-old female with Polycystic Ovary Syndrome (PCOS), T2DM, acanthosis nigricans on the anterior and posterior neck, axillae, inner thighs, and groin, hirsutism, poor glycemic control and hyperinsulinemia	HAIR-AN	Liraglutide	Liraglutide subcutaneous injection: 0.3 mg, once daily and metformin: 0.5 g, four times daily (2 g total per day). Total treatment time: 13 months.	The patient experienced normalized menstrual cycles and improvement of acanthosis nigricans on visual inspection. The patient lost 9.5 kg, had normalization of liver and lipid panels and decrease of insulin and C-peptide levels throughout follow-up visits.
Malavazos et al. (2023) [10]	Case Report	50-year-old female with obesity, T2DM and psoriasis diagnosed in 2006. The patient has had unsuccessful trials of ixekizumab, secukinumab and guselkumab. The patient was not on any other medications at the start of the trial.	Psoriasis	Semaglutide	0.25 mg per week subcutaneously for 4 weeks and then 0.5 mg per week until a maintenance dose of 1 mg per week was reached at 16 weeks. Total treatment time: 10 months.	DLQI score dropped from 20 to 1, Disease Activity in Psoriatic Arthritis (DAPSA) dropped from 31 to 4 and PASI score dropped from 12 to 0.2. Notable subjective improvement of hand lesions noted. BMI decreased from 30.4 kg/m² to 22.6 kg/m², and HOMA-IR was reduced from 3.32 to 0.84. C-reactive protein (CRP) dropped from 4.3 to <0.5, IL-6 dropped from 4.9 to 3.4 and Erythrocyte Sedimentation Rate (ESR) dropped from 21 to 12.
Nowowiejska et al. (2023) [11]	Case Report	34-year-old female with a history of psoriasis and insulin resistance.	Psoriasis	Liraglutide	Not reported. Treatment period: 2 months.	New onset and provocation of erythematous scaly lesions on face, scalp, mammary and genital regions.
Nicolau et al. (2023) [12]	Prospective cohort study	20 subjects with psoriasis and obesity without T2DM use or on any active anti-obesity drug. All patients had been on either a biological or phototherapy regimen for psoriasis for at least three months prior to the start of the study.	Psoriasis	Liraglutide	Liraglutide dose of 0.6 mg per week subcutaneously for the first week increasing up to 3 mg towards the end of the trial. Total treatment time: three months. Notes: Subjects also received diet and exercise with a suggested average of 500 kcal/day reduction from calculated baseline metabolic rate and a minimum of 150 minutes of aerobic exercise per week.	Mean PASI score dropped from 10 to 5 (p<0.001), mean Visual analog scale (VAS) score dropped from 4.1 to 2.3 (p=0.009) and mean DLQI score dropped from 13 to 6 (p=0.009). Mean BMI decreased from 38.9 to 36.4 (p = 0.003), and mean HOMA-IR decreased from 4.4 to 3.2.
Barry et al. (2024) [13]	Case report	60-year-old women with Hailey-Hailey disease with a 40-year history of the disease refractory to treatment. The patient was recently diagnosed with T2DM. Patient has lesions on neck, axillae, and inguinal and abdominal folds.	Hailey-Hailey disease	Liraglutide	Liraglutide once daily subcutaneous injection of 1.8 mg. Total treatment time: 6 months.	Near complete resolution of lesions on neck, axillae, and inguinal and abdominal folds. The patient reported resolution of associated pain as well.
Nicolau et al. (2024) [14]	Prospective cohort study	14 subjects with HS and obesity without T2DM. All patients had been on either a biological or topical antibiotic regimen for psoriasis for at least three months prior to the start of the study.	Hidradenitis suppurativa	Liraglutide	Liraglutide dose of 0.6 mg per week subcutaneously for the first week increasing up to 3 mg within 5 weeks of starting the trial. Total treatment time: three months. Notes: Subjects also received diet and exercise with a suggested average of 500 kcal/day reduction from calculated baseline metabolic rate and a minimum of 150 minutes of aerobic exercise per week.	Mean Hurley score dropped from 2.6 to 1.1 (p=0.002), mean VAS score dropped from 5.6 to 3.2 (p=0.003) and mean DLQI score dropped from 12.3 to 9.7 (p=0.04). Mean BMI dropped from 39.3 to 35.6 (p=0.002).
Anatolieva et al. (2024) [15]	Case Report	38-year-old woman with metabolic syndrome, autoimmune thyroiditis and a two-year history of persistent pruritic firm bumps on the posterior neck and occipital region of the scalp	Acne Keloidalis Nuchae	Liraglutide	Levothyroxine 125 mcg per day, metformin 2000 mg per day, liraglutide 0.6 mg per day subcutaneously and spironolactone 50 mg per day. Treatment period: 1 year. Note: patients in study reported poor treatment adherence.	There was a reported reduction in fibrotic plaque size and resolution of acne lesions. There was no improvement of acanthosis nigricans lesions or acrochordons in nuchal and axillary folds. The patient reported a reduction in weight and resolution of “bradypsychic state” and overall well-being.
Gordon et al. (2024) [16]	Case report	57-year-old man with a BMI of 33.45 kg/m2, androgenic alopecia, acanthosis nigricans and insulin resistance. Prior to treatment, there was reported increased hair shedding, vertex scalp with decreased hair density and widened part and bitemporal scalp with mild hair recession.	Androgenic alopecia	Tirzepatide	Tirzepatide 2.5 mg every 7 days for 3 months. The dose was then increased to 5 mg weekly for 6 months before moving to a final dose of 7.5 mg weekly. Total treatment time: 1 year.	Marked improvement of hair loss at vertex scalp and bitemporal scalp with increased hair regrowth noted at 6 months. The patient lost 30 lbs, and HOMA-IR decreased from 3.2 to 1.7.
Chan et al. (2024) [17]	Case report	Female patient in her 20s with a 10-year history of severe HS, PCOS, T2DM and obesity. The patient failed prior therapies including adalimumab and was on infliximab over the course of the trial.	Hidradenitis suppurativa	Tirzepatide	Starting course of tirzepatide 2.5 mg/0.5 mL once a week with an increase to 7.5 mg/0.5 mL once a week over the course of 3 months.	The patient reported continued boils but reduced inflammation. DLQI score decreased from 14 to 3, VAS decreased from 3 to 1 and the HS-PGA score decreased from 3 to 2. Hidradenitis Suppurativa Clinical Response (HiSCR) scores were notable for decreases of 1 abscess to 0 and 4 inflammatory nodules to 3 without draining fistulas.
Morrissette et al. (2024) [18]	Case report	Male patient in his 40s presented with a 30-year history of Folliculitis Decalvans. The patient had failed multiple topical, oral, and intralesional therapies.	Folliculitis Decalvans	Tirzepatide	Weekly subcutaneous tirzepatide injections starting at 2.5 mg and titrated to 12.5 mg weekly. Total treatment time: nine months. Note: At the end of the course of treatment, the patient’s dosage was tapered down but following a fare up in symptoms, the dose was restored to 7.5 mg weekly.	The patient noted reductions in pain, drainage, flares and prominent hair regrowth. BMI decreased from 41.36 to 33.63. There appeared to be a decrease in erythema and fluctuance on presentation.
Hill et al. (2024) [19]	Retrospective cohort study	75,178 Patients with HS between 18 to 90 years old with a BMI of > 27 kg/m 2 with at least 1 weight-related condition (high cholesterol, high blood pressure, and type 2 diabetes). Two cohorts were formed and matched with a subset with semaglutide exposure and one the other without.	Hidradenitis suppurativa	Semaglutide	All patients evaluated, whether exposed to semaglutide or not were on some combination of biologics or antibiotics.	In comparison to the control group, there was decreased use of antibiotics (Risk Ratio (RR): 0.758, Confidence Interval (CI): 0.732-0.785), steroids (RR: 0.839, CI: 0.811-0.868), and visits to the emergency department (RR: 0.715, CI: 0.681-0.751). No difference was observed for biologic usage (RR: 0.983, CI: 0.862-1.122). HiSCR scores were also decreased.
Posada et al. (2024) [20]	Retrospective cohort study	45 subjects with HS. 29 subjects were Caucasian, 10 were Black and 2 were Asian. Thirteen patients had Hurley stage I HS, 27 had Hurley stage II HS and 5 patients had Hurley stage III HS.	Hidradenitis suppurativa	Semaglutide	The mean Semaglutide starting dose was 0.52 mg/week which on average increased to 1.11 mg/week and 1.36 mg/week by 6 and 12 months respectively. Total treatment time: 12 months. Patients were on various differing combinations of oral therapies and biologics.	Of the 45 patients treated, 27 showed improvement and 18 did not. Of the patients that showed improvement, 9 were classified as HS I, 16 HS II and 2 at HS III. Of those that did not show improvement, 4 were in HS I, 11 were in HS II and 3 were in HS III. The HS findings were not statistically significant. In a breakdown by race, 19/29 of the Caucasian patients showed improvement, 4/10 of the Black patients showed improvement and 2/2 of the Asian patients showed improvement (p=0.31). Patients that showed improvement had a BMI of 40.9, an Hemoglobin A1c (HbA1c) of 6.9% and a gain of 10.6 lbs on average. Those that did not show improvement had a BMI of 40.8, an HbA1c of 7.5% and a gain of 9.3 lbs on average. These findings were not statistically significant.
Petković-Dabić et al. (2025) [21]	Randomized-controlled trial	31 participants between 18-70 years of age with psoriasis, T2DM and on a metformin regimen.	Psoriasis	Semaglutide	Control group: Maximally tolerated metformin dose. Experimental group: Maximally tolerated metformin dose with 1.0 mg/patient/week of subcutaneous semaglutide injections. Total treatment period: 12 weeks.	In the control group, median PASI scores decreased from 20.6 to 15.9 (p = 0.03) and mean DLQI scores decreased from 10.1 to 8.1 (p = 0.007). Mean BMI decreased from 36.3 to 34.8 (p < 0.001). IL-6 decreased from 5.6 to 2.3 pg/mL (p = 0.1), IL-23 decreased from 87.5 to 51.6 pg/mL (p = 0.1), and mean CRP decreased from 9.6 to 7.6 mg/L (p = 0.5). In the intervention group, median PASI scores decreased from 21 to 10 (p = 0.002) and median DLQI scores decreased from 14 to 4 (p = 0.002). Mean BMI decreased from 33.0 to 30.7 (p = 0.001). Mean CRP decreased from 3.8 to 1.9 (p = 0.01), IL-6 decreased from 3.5 to 2.8 (p = 0.05), and IL-23 decreased from 51.9 to 41.2 pg/mL (p = 0.2).
Chen et al. (2025) [22]	Case report	A 14-year-old female with a history of childhood-onset Congenital Linear Scleroderma (CLS), previously managed with steroids, methotexate (MTX), and mycophenolate mofetil (MMF), presented with progressive erythema, atrophy, and subcutaneous fat loss despite multiple immunosuppressive regimens.	Congenital Linear Scleroderma	Semaglutide	Total treatment period: 7 months	The patient noted improved mobility and reduced skin hardness in her left arm, with no disease flares. Physical exam at follow up showed only mild atrophy and faint discoloration, with supple skin and no significant erythema.

Literature search

Psoriasis

In randomized trials, cohort studies, and case reports, GLP-1RA therapy was associated with improvements in PASI and DLQI scores, with some case reports noting near-complete lesion resolution. Randomized trials and cohort studies generally showed greater PASI and DLQI reductions in GLP-1RA groups compared with controls, though statistical significance varied. A randomized trial of 20 glucose-tolerant patients found no significant differences between groups, whereas the other studies, including additional randomized controlled trials, retrospective cohort studies, and case reports encompassing approximately 85 patients, demonstrated meaningful improvements. These clinical benefits often coincide with reductions in BMI, insulin resistance (IR), and systemic inflammation, including decreases in CRP, IL-6, IL-17, and IL-23. An isolated case report described worsening of psoriasis with liraglutide. Reported adverse effects were generally mild, primarily gastrointestinal; one patient in one of the controlled trials developed a rash with liraglutide, but no other cutaneous adverse effects were reported across the studies aside from the psoriasis case report.

Hidradenitis Suppurativa

In case reports and cohort studies, GLP-1RA therapy was associated with reductions in lesion burden, drainage, and flares, along with decreased pain and improved quality of life (DLQI, VAS, HS-PGA). Objective outcomes included reduced need for antibiotics, steroids, and emergency care. Benefits were most pronounced in patients with obesity or type 2 diabetes. No cutaneous adverse events were reported.

Hyperandrogenism, Insulin Resistance, and Acanthosis Nigricans (HAIR-AN) Syndrome

In the two available studies, GLP-1RA therapy was associated with normalization of menstrual cycles, diminished acanthosis nigricans, reduction in hirsutism, and improvements in serum testosterone. No cutaneous adverse events were noted.

Hailey-Hailey Disease

In a single case report by Barry et al., a patient with refractory Hailey-Hailey disease achieved near-complete resolution of lesions and complete pain relief. No objective clinical markers were reported, and no adverse events occurred during treatment.

Acne Keloidalis Nuchae

In a single case report by Anatolieva et al., a patient with acne keloidalis nuchae experienced a reduction in fibrotic plaque size and resolution of acne lesions, with no change in coexisting acanthosis nigricans or acrochordons. No adverse events were reported.

Folliculitis Decalvans

In a single case report by Morrissette et al., a patient with Folliculitis Decalvans showed reductions in pain, drainage, and flares, along with prominent hair regrowth and decreased erythema and fluctuance.

Androgenic Alopecia

In a single case report by Gordon et al., a 57-year-old man with androgenic alopecia experienced marked improvement in hair loss at the vertex and bitemporal scalp, with visible regrowth at six months. No adverse events were reported.

Congenital Linear Scleroderma 

In a single case report by Chen et al., a 14-year-old female with childhood-onset congenital linear scleroderma, refractory to multiple immunosuppressive regimens, saw improvements in mobility and reduced skin hardness without flares following semaglutide use. 

Discussion

Although the strength of evidence varies across conditions, existing reports collectively suggest that GLP-1RAs may modulate dermatologic disease via overlapping metabolic and immunologic pathways.

Metabolic Pathways (Weight Loss, Insulin Resistance, Hyperinsulinemia)

The most consistently observed pathway by which GLP-1RAs may improve dermatologic disease is through their weight loss-mediated effects. Obesity is widely recognized as a chronic, low-grade inflammatory state, driven by adipokines (e.g., leptin, resistin) and cytokines (e.g., TNF-α, IL-6, CRP), which exacerbate immune dysregulation and barrier disruption [23-25].

In psoriasis, obesity is strongly associated with incidence, severity, and treatment resistance [26]. Weight reduction through lifestyle interventions improves PASI, DLQI, and treatment outcomes, as demonstrated in both meta-analyses and randomized controlled trials [27-30]. GLP-1RAs achieve weight loss through satiety, reduced caloric intake, and improved insulin sensitivity, providing a plausible explanation for the clinical improvements observed in reported studies [31]. Several studies of modern biologics also indicate that higher BMI is linked to diminished responses and that weight reduction may enhance outcomes [32,33].

In HS, obesity contributes both mechanically (skin folds, friction, humidity) and immunologically (adipokine-driven inflammation) [34-39]. Excess adiposity promotes cytokine release, impairs wound healing, and drives follicular rupture [35,40]. Weight loss via bariatric surgery can improve HS, though rapid postoperative weight loss may paradoxically worsen lesions through nutritional deficiencies [41]. Importantly, GLP-1RAs achieve gradual weight reduction, with studies reporting improvements in insulin resistance, CRP, and homocysteine, as well as clinical gains in Hurley stage and DLQI, without the nutritional deficiencies seen after bariatric surgery.

Insulin resistance (IR) represents a common mechanistic link. In HAIR-AN syndrome, hyperinsulinemia drives ovarian androgen excess, follicular disruption, and keratinocyte proliferation, manifesting as hirsutism, acne, alopecia, and acanthosis nigricans [42,43]. GLP-1RAs improve IR, reduce insulin and androgens, and may ameliorate features of HAIR-AN, though use remains investigational [44-46]. Both psoriasis and HS are associated with IR, likely driven by chronic inflammation and cytokine dysregulation [47-50]. One psoriasis randomized controlled trial study of liraglutide use in obese but glucose-tolerant patients showed no improvement in PASI or DLQI, despite metabolic benefit, suggesting glycemic status may influence therapeutic response [2].

In AGA, hyperinsulinemia may upregulate 5-α reductase and accelerate follicular miniaturization, supported by murine data though limited human confirmation [51,52]. In acne keloidalis nuchae, obesity itself is not a driver, but associations with metabolic syndrome suggest potential systemic benefits of GLP-1RAs without direct cutaneous effects [53,54].

Direct Immunologic and Cutaneous Pathways

Beyond weight loss, GLP-1RAs modulate inflammatory networks central to skin disease. They suppress IL-1β, IL-6, IL-17, and TNF-α, paralleling mechanisms of commonly used biologics [55-57]. Because these cytokines are central to the pathogenesis of immune-mediated dermatoses, their downregulation may help explain observed clinical responses, though most evidence derives from metabolic or animal models rather than skin-specific studies.

In psoriasis, GLP-1RAs have been shown in both basic science models and psoriasis-specific studies to reduce IL-17, attenuate NF-κB signaling, and redistribute invariant natural killer T (iNKT) cells from plaques into circulation [57-59]. Since IL-17 and TNF-α are central to psoriasis pathogenesis, these changes may help explain clinical improvements, though much of the mechanistic detail still derives from preclinical or systemic work. A role for dermal γδ T-cell modulation has also been proposed but remains speculative [60]. Notably, these immunologic effects overlap with pathways targeted by psoriasis biologics, suggesting GLP-1RAs may provide adjunctive benefit in obese patients where biologic responses are attenuated. At the same time, paradoxical psoriasis worsening has been observed in isolated cases, underscoring the complexity of cytokine balance [11].

In hidradenitis suppurativa (HS), laboratory studies suggest GLP-1RAs downregulate TNF-α, IL-17, and IL-23 signaling and inhibit chemokine-driven T-cell trafficking [57-64]. They also promote macrophage polarization toward reparative M2 phenotypes, enhance keratinocyte migration, support angiogenesis, and improve keratinocyte cohesion via PI3K/Akt signaling [65-67]. Together, these immunologic and barrier-restorative actions align with HS pathogenesis and broader cutaneous repair mechanisms and may help explain reported clinical improvements, with potential relevance to other disorders such as Hailey-Hailey disease.

Inflammatory and Fibrotic Dermatoses

Beyond psoriasis and HS, related cytokine pathways may also be relevant in other conditions. Localized scleroderma has been linked to elevated TNF-α and IL-6 [68-70]. Folliculitis decalvans involves dysregulation of IL-1β, IL-8, and IL-17, reflecting a neutrophil-driven inflammatory response [71]. Keloid formation, including acne keloidalis nuchae, has been associated with IL-17-mediated fibrotic inflammation [72]. While GLP-1RAs have not been directly studied in these disorders, their ability to suppress these cytokines in preclinical and systemic studies provides a biologically plausible rationale for further investigation.

Microvascular Perfusion and Receptor Expression

GLP-1 receptor expression has been demonstrated in cutaneous immune infiltrates, including psoriatic plaques and allergic contact dermatitis lesions, supporting biological plausibility for direct immune modulation [73,74]. Additionally, GLP-1RA-associated improvements in microvascular perfusion may be pertinent to follicular disorders such as androgenic alopecia and folliculitis decalvans, where perifollicular inflammation and vascular supply influence disease activity [75].

**Figure 2:**
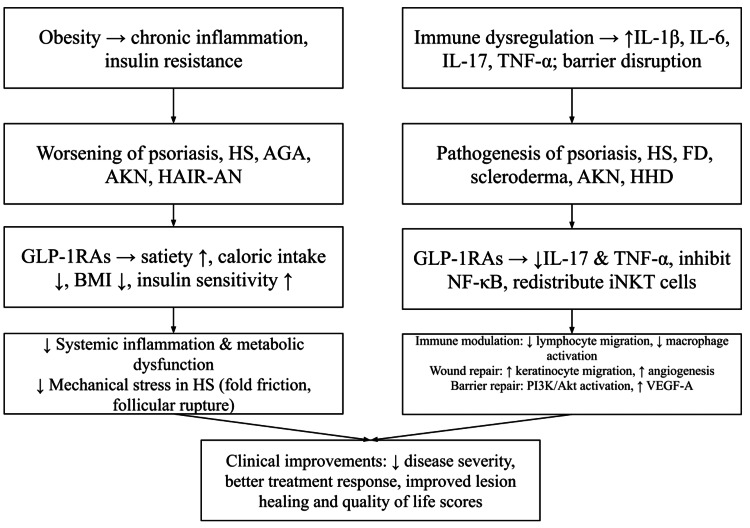
Proposed Dual Mechanisms of GLP-1 Receptor Agonists in Dermatologic Disease HS: hidradenitis suppurativa, AGA: androgenic alopecia, HAIR-AN: Hyperandrogenism, insulin resistance, and acanthosis nigricans, AKN: acne keloidalis nuchae, FD: folliculitis decalvans, HHD: Hailey-Hailey Disease, GLP-1RA: glucagon-like peptide-1 receptor agonist, BMI: body mass index The image is created by the author.

While the evidence remains early, our review highlights a theorized dual action of GLP-1RAs mediated through weight reduction as well as direct immune and epithelial effects. Figure 2 summarizes these proposed mechanisms.

Limitations

This review has several limitations that warrant consideration. Many of the included studies involved the concurrent use of other pharmacologic agents to manage metabolic comorbidities, which may confound the dermatologic outcomes attributable solely to GLP-1 receptor agonist (GLP-1RA) therapy. In addition, the majority of patients studied had obesity or insulin resistance, so data on GLP-1RA use in glucose-tolerant or non-obese populations remain limited. A further limitation is the absence of head-to-head comparisons between GLP-1RAs, which restricts conclusions about whether specific agents provide greater dermatologic benefit. Notably, among the psoriasis studies, one trial in glucose-tolerant patients reported no improvement, and another reported worsening, both involving liraglutide. Risk of bias assessments indicated that randomized trials and observational cohorts were of adequate quality, and case reports largely met JBI criteria but remain anecdotal. Because the literature was heterogeneous, different tools were required for each design, which limited direct comparison across study types. Finally, while GLP-1RAs show promise in dermatologic contexts, their reliable use by dermatologists must account for their systemic safety profile. Known adverse effects, including pancreatitis and gastrointestinal symptoms, as well as reported cutaneous reactions such as dermal hypersensitivity, eosinophilic panniculitis, pyoderma gangrenosum, and bullous pemphigoid, highlight the need for cautious clinical consideration and further investigation [76].

## Conclusions

Our review highlights the promising therapeutic potential of GLP-1 receptor agonists in treating a wide range of dermatologic conditions associated with metabolic dysfunction, such as psoriasis and HS, as well as conditions with emerging links to metabolic health, such as androgenic alopecia. The reviewed literature suggests that these benefits are plausibly mediated by both weight loss-related reductions in systemic inflammation, insulin resistance, and mechanical stress, as well as potential direct immunomodulatory and cutaneous effects. As GLP-1RAs address underlying metabolic abnormalities and pathologic immunologic signaling, they may provide a novel, dual-action approach to treating skin diseases linked to metabolic disturbances. Taken together, these findings suggest a potential shift in how dermatologists approach the management of skin conditions associated with obesity, insulin resistance, and systemic inflammation, though further mechanistic and clinical studies will be needed to clarify the extent of these effects.
